# MSCs-Derived Decellularised Matrix: Cellular Responses and Regenerative Dentistry

**DOI:** 10.1016/j.identj.2024.02.011

**Published:** 2024-03-16

**Authors:** Suphalak Phothichailert, Shirel Samoun, Benjamin P. Fournier, Juliane Isaac, Sindy Cornelia Nelwan, Thanaphum Osathanon, Nunthawan Nowwarote

**Affiliations:** a Center of Excellence for Dental Stem Cell Biology, Faculty of Dentistry, Chulalongkorn University, Bangkok, Thailand; b Centre de Recherche des Cordeliers, Université Paris Cité, Sorbonne Universite, INSERM UMRS1138, Molecular Oral Pathophysiology, Paris, France; c Department of Oral Biology, Faculty of Dentistry, Université Paris Cité, Paris, France; d Department of Pediatric Dentistry, Faculty of Dental Medicine, Universitas Airlangga, Airlangga, Indonesia; e Department of Anatomy, Faculty of Dentistry, Chulalongkorn University, Bangkok, Thailand

**Keywords:** Regenerative dentistry, Extracellular matrix, Decellularisation, Mesenchymal stem cell, Cell proliferation, Cell differentiation

## Abstract

The decellularised extracellular matrix (dECM) of *in vitro* cell culture is a naturally derived biomaterial formed by the removal of cellular components. The compositions of molecules in the extracellular matrix (ECM) differ depending on various factors, including the culture conditions. Cell-derived ECM provides a 3-dimensional structure that has a complex influence on cell signalling, which in turn affects cell survival and differentiation. This review describes the effects of dECM derived from mesenchymal stem cells (MSCs) on cell responses, including cell migration, cell proliferation, and cell differentiation *in vitro*. Published articles were searched in the PubMed databases in 2005 to 2022, with assigned keywords (MSCs and decellularisation and cell culture). The 41 articles were reviewed, with the following criteria. (1) ECM was produced exclusively from MSCs; (2) decellularisation processes were performed; and (3) the dECM production was discussed in terms of culture systems and specific supplementations that are suitable for creating the dECM biomaterials. The dECM derived from MSCs supports cell adhesion, enhances cell proliferation, and promotes cell differentiation. Importantly, dECM derived from dental MSCs shows promise in regenerative dentistry applications. Therefore, the literature strongly supports cell-based dECMs as a promising option for innovative tissue engineering approaches for regenerative medicine.

## Graphical abstract

A summary of decellularisation to recellularisation illustrated the process of generating natural biomaterials and their impact on cellular responses.

**Figure:**
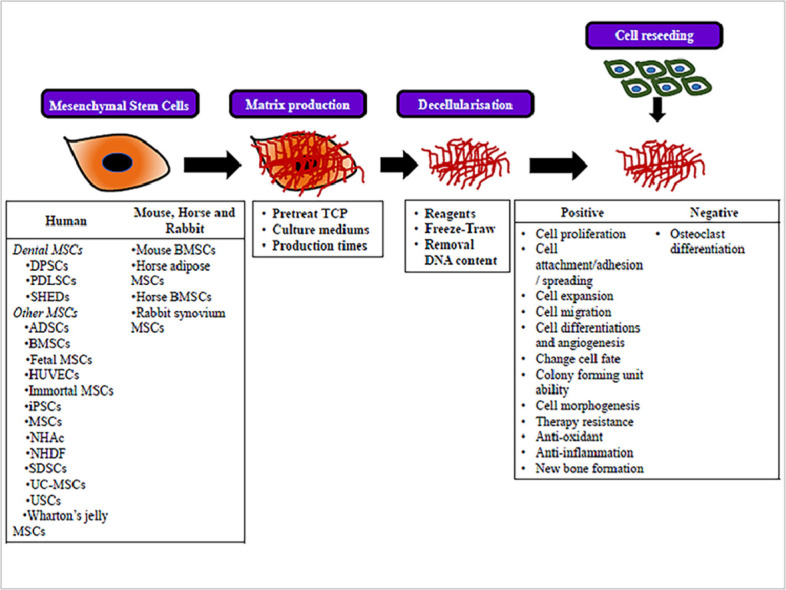

## Introduction

The extracellular matrix (ECM) serves as a scaffold, offering support to cells and tissues. It affects cell responses through interactions with receptors and acts as a reservoir for signalling molecules, indirectly governing cell behaviour. Its properties are vital for development, growth, and regeneration.^1^ The ECM substrate is widely used as a biomaterial in regenerative medicine and tissue engineering. To create an ECM scaffold, genetic materials (eg, DNA) are removed using techniques such as chemical, mechanical, or enzyme digestion methods. This process leads to the decellularised extracellular matrix (dECM). Studies involving animal models have shown successful use of dECM to regenerate damaged kidneys, liver, heart, lungs, and other organs.^2^, ^3^, ^4^

The purpose of dECM utilisation is to create an appropriate microenvironment for cells to proliferate and differentiate, subsequently restoring damaged or diseased tissues. Critical steps in the production of dECM are considered, including the source of ECM, the decellularisation process, and postmodification. Therefore, investigations of the biological properties of the dECM process using various techniques are required. This review describes a brief overview of cellular responses to dECM derived from cultured mesenchymal stem cells (MSCs) and strategies for application in regenerative medicine.

## Material and methods

A PubMed database search (Jan 2000-Dec 2022) used keywords: MSCs [All Fields] AND decellularization [All Fields] AND cell culture techniques [All Fields]. Article titles and abstracts are reviewed. Inclusion: (1) MSC-derived ECM, (2) decellularization, and (3) dECM production with culture systems and supplements.

## Results and discussions

From PubMed, 124 articles were found with assigned keywords. Among the 41 meeting criteria, 4 articles related to regenerative dentistry were identified. These selected articles formed the basis of this narrative review.

### Classification of the ECM

The ECM proteins can be categorised into several main categories: glycoproteins, proteoglycans, and fibrous proteins, based on their molecular structure. The concept of the ECM matrisome has been introduced, consisting of 2 groups: the core matrisome proteins (collagens, glycoproteins, and proteoglycans) and matrisome-associated proteins (ECM regulators and secreted factors) (Table 1). Mass spectrometry is used to quantify peptides and proteins, allowing for protein identification.^5^ Matrisome is a proteomic database available for humans, mice, and nematodes (*Caenorhabditis elegans*) (http://matrisomeproject.mit.edu).^6^^,^^7^ Comprehending composition and function aids targeted interventions, modulating the ECM for tissue repair and regeneration.^8^ Essentially, the matrisome represents the complete set of ECM proteins and associated factors synthesised and secreted by cells, playing crucial roles in cell adhesion, signalling, and other processes.

**Table 1:** Classification of extracellular matrix proteins by protein structural and proteomic matrisome database.

**Classical ECM**		
Glycoproteins	Proteins that have attached oligosaccharide chains to the amino acid	eg, *fibronectins, laminins, vitronectin, thrombospondins, tenascins, entactins, nephronectin, fibrinogen*
Proteoglycans	The protein comprises attached sulphated GAGs linked to the core proteins.	eg, *collagens and elastin*
Fibrous proteins	Proteins composed of polypeptide chains that exhibit elongated and fibrous structures or sheet-like structures.	eg, *GAG chains, chondroitin sulphate, dermatan sulphate, heparan sulphate, keratan sulphate, and syndecans*
**ECM matrisome database**		
**Core matrisome proteins**		
Glycoprotein	eg, *fibrins, laminins, tenascins, thrombospondins, fibrins, fibulins, and others.*	
Proteoglycans	*-*	
Collagens	*Including transmembrane collagens*	
**Matrisome-associated proteins**		
ECM-affiliated proteins	*- Proteins that could be considered ECM proteins (*eg, *mucins, c-type lectins, syndecans, glypicans)* *- Proteins that appear repeatedly in ECM-enriched preparations (*eg, *annexins, galectins)* *- Secreted factors and associated with solid-phase complexes (*eg, *semaphorins and their homologous receptors, plexins, collagen-related proteins and their homologous)*	
ECM regulators	*- ECM crosslinking (*eg, *lysin oxidases, transglutaminases)* *- ECM-modifying enzymes (*eg, *sulphatase, extracellular kinases)*	
Secreted factors	*Protein and other cytokines,* eg, *transforming growth factor β, bone morphogenetic*	

ECM, extracellular matrix; GAGs, glycosaminoglycans.

### Decellularisation processes

Cell culture-derived dECM is a naturally occurring biomaterial obtained by removing cellular components from isolated culture conditions. The decellularisation process aims to retain the ECM's structure and bioactive molecules while eliminating cellular elements. The surface chemistry of the substrate appears to have no influence on the microarchitecture or composition of the cell culture-derived dECM. Decellularisation techniques involve the use of chemical or detergent solutions, sometimes coupled with mechanical methods, to completely remove cellular components, preserving the ECM structures.^9^, ^10^, ^11^, ^12^ During the process, several limitations occur, including: (1) the ECM protein loss, (2) the decellularisation is incomplete due to high cellular confluence, (3) the chemicals’ concentration and incubation time varies, (4) ECM detaches from the substrate before recellularisation, and (5) the technique should not cause cytotoxicity or immunological response.^11^, ^12^, ^13^ Several decellularisation methods have been developed for the production of MSC-derived dECM, including chemical, enzymatic, and physical methods. Various decellularised formulas have been developed (Table 2). For example, decellularisation with 2M potassium chloride (KCL) and 0.2% Triton X-100 yields ECM rich in laminin.^14^ The use of 1% Triton X-100 with 20 mM NH_4_OH in PBS preserves the ECM structure.^15^ A combination of Triton X-100 and freeze-thaw cycles leads to the best-preserved structure and components of the ECM.^11^ Furthermore, 1% to 5% of sodium dodecyl sulphate (SDS) slightly affects the ECM and increases collagen.^16^ DNases are essential to remove the remaining DNA content in dECM.^17^, ^18^, ^19^ Optimised processes can be done by adjusting time, chemical agent, temperature, and physical application methods (ie, agitation). MSC-derived dECM requires optimising numerous parameters, including:

1. ECM structure preservation is crucial for tissue's biomechanical properties.
2. The cellular elements (ie, DNA, RNA, and debris) must be efficiently removed, but the ECM proteins must be retained.
3. The dECM should be nontoxic and nonimmunogenic. It should also support cell attachment, proliferation, and differentiation.
4. Uniform dECM scaffold properties must be ensured for consistency and reproducibility.
5. Decellularisation must be scalable for large-scale dECM scaffold production in clinical use.

**Table 2:** (A) Decellularisation solutions.

Reagents	Removal DNA content	Number of studies
0.1% Triton X-100 containing 10 mM NH_4_OH and 5 mM EDTA in PBS	DNase and RNase A	1 (84)
0.25% Triton X-100 containing 10 mM NH_4_OH in PBS	DNase and RNase A	1 (39)
0.5% Triton X-100 solution containing 20 mM NH_4_OH in PBS	-	15 (10, 16, 30, 32, 38, 47, 48, 50, 51, 56, 59, 67, 85-87)
0.5% Triton X-100 solution containing 20 mM NH_4_OH in PBS	DNase	14 (13, 19, 27-29, 35, 40, 43, 45, 46, 49, 53, 58, 88)
0.5% Triton X-100 solution containing 20 mM NH_4_OH in PBS	DNase and RNase A	1 (18)
0.5% Triton X-100 solution containing 20 mM NH_4_OH in PBS	DNase, RNase A, and lipase or urea	2 (34, 54)
0.5% Triton X-100 solution containing 25 mM NH_4_OH in PBS	DNase	1 (36)
0.5% Triton X-100 in PBS and freeze-thaw cycle	-	1 (11)
1% Triton X-100 solution in dH_2_O or containing 20 mM NH_4_OH in PBS	DNase	1 (15)
1% Triton X-100 solution containing 25 mM NH_4_OH in PBS	DNase	1 (17)
25 mM NH_4_OH in PBS and freeze-thaw cycle	-	1 (12)
Double-distilled water (DDW) containing 20 mM NH_4_OH	DNase	2 (41, 42)

PBS, phosphate-buffered saline.

Therefore, the decellularised process should ideally retain the native structure and composition while minimising immunogenicity and preserving bioactivity. Additionally, the process of obtaining dECM involves a variety of methods. Each method presents specific advantages and disadvantages, as shown in Table 2B.

**Table 2:** (B) The decellularisation methods present specific advantages and disadvantages.

Methods	Advantages	Disadvantages
Chemicals	• *Relatively simple and straightforward process applicable to cell culture systems.* • *Can be used with a variety of cell types.* • *Preserves the structural integrity of the ECM*	• *Damage to certain ECM components.*
Enzymatic digestion	• *Essential to remove DNA content.*	• *May affect the bioactivity of growth factors.*
Chemicals and enzymatic digestion	• *Completely remove intracellular components.*	• *May affect both ECM component and bioactivity of growth factors.*
Freeze-thaw cycling	• *Relatively simple and cost-effective.* • *A gentle method, preserving some ECM structure.*	• *May not be as effective in fully removing intracellular components.* • *Effectiveness might vary based on cell and tissue types.*

ECM, extracellular matrix.

### The effect of the microenvironment on cellular-derived ECM production

The microenvironment has a significant effect on ECM production by regulating the behaviour of cells responsible for producing and depositing ECM molecules. The microenvironment comprises physical and biological factors that influence ECM production, altering composition, structure, and function.^20^^,^^21^ In regard to physical factors, cyclic loading strain raised elastin and collagen levels in smooth muscle cells, resulting in an improvement in tissue organisation.^22^ Meanwhile, the mechanical loading force enhanced the synthesis of ECM while seeding on scaffold material.^23^ However, further investigation is needed to understand the impact of mechanical loading on MSCs’ ECM production and its components, as well as to identify the optimal conditions for ECM production.

Biological factors such as growth factors and cytokines also play a crucial role in regulating the production of ECM. Transforming growth factor-β (TGF-β)^24^ and bone morphogenetic protein (BMP)^25^ stimulate ECM synthesis by promoting the differentiation of precursor cells into ECM-producing cells and increasing the production of ECM proteins. Similarly, cytokines such as interleukin-1 (IL-1) and tumour necrosis factor-α (TNF-α) enhance the production of ECM-degrading enzymes, leading to the breakdown of the ECM.^26^ Culture conditions play a vital role in producing ECM that influences desired cell responses. Under a chondrogenic medium, dECM enhances MSCs viability, spreading, and proliferation, redifferentiation, and anti-inflammatory properties. Conversely, dECM from an ascorbic acid-supplemented medium or osteogenic medium culture promotes the highest calcium accumulation.^13^^,^^27^, ^28^, ^29^ Three-dimensional dECM provides a specific environment for specific cell type to retain their differentiation ability but does not affect cell proliferation.^30^

Using dECM to mimic the *in vivo* extracellular microenvironment has been shown to be a useful strategy to stimulate cell proliferation and survival.^31^ The 3-dimensional structures of the ECM support cell growth and serve as a reservoir of growth factors and cytokines that control cell destiny and function.^19^^,^^31^^,^^32^ This complexity contributes to the formation of optimal niches for cells to reside in. Furthermore, 3-dimensional culture conditions could lead to an ECM that physiologically resembles native tissue more closely than those derived from the 2-dimensional culture system.^33^ Together, these results emphasise the significance of microenvironment and culture conditions in the bioactive characteristics of dECM.

### Effect of dECM derived from MSCs on cellular responses

MSCs possess self-renewal and proliferation abilities, contributing to regeneration through direct differentiation towards specific cell types and the secretion of regenerative-related factors. MSCs can be extracted from bone marrow, umbilical cord, fat, cartilage, urine, and dental tissues. The *in vitro* expansion of MSCs has limitations, such as cell senescence and reduced differentiation capacity. Studying dECM from diverse MSC sources is crucial for creating targeted microenvironments, aiding tissue regeneration.

MSC-derived dECM affects cellular responses diversely, for example, by promoting cell adhesion, migration, and proliferation, as well as modulating cell differentiation and immune responses. MSC-derived dECM exhibits lower rejection risk by using the patient's own cells. Nevertheless, some studies show that dECM from MSCs triggers an immune response in animals, leading to an increased production of cytokines and immune cell infiltration.^12^ However, this immune response does not appear to affect the functional properties of the dECM.

#### Adipose-derived stem cells

Adipose-derived stem cells (ADSCs) are easily accessible and widely used in regenerative medicine due to their self-renewing and versatile nature. dECM-ADSCs exhibit more collagen and glycosaminoglycans when cultured in an osteogenic differentiation medium.^34^ Additionally, dECM from ADSCs plays a crucial role in regulating retinal progenitor cell proliferation and differentiation, holding promise for enhancing the effectiveness of retinal progenitor cell treatment in retinal degenerative diseases.^35^ The microstructure and native constituents preserved in the dECM could provide a useful platform for studying the role of ECM in wound healing.^17^ Furthermore, it also affects pathologic changes by supporting repopulation in cancer cells during chemotherapy treatment.^36^

#### Bone marrow MSCs

Bone marrow MSCs (BMSCs)-derived dECM contains multiple proteins that dramatically promote the proliferation, migration, and differentiation of MSCs.^37^, ^38^, ^39^, ^40^, ^41^, ^42^, ^43^ dECM derived from foetal BMSCs promotes MSCs with a higher proliferative and osteogenic capacity compared to adult BMSCs-dECM.^44^^,^^45^ Furthermore, BMSCs-derived dECM suppressed osteoclastogenesis, implying positive anabolic effects on bone regeneration. dECM derived from BMSCs could benefit in clinical use by modulating bone remodelling and promoting bone tissue engineering.^46^ BMSCs-dECM enhances chondrogenesis of chondrocytes both *in vitro* and *in vivo*^47^ and modulates human umbilical vein endothelial cell (HUVECs) responses in the vascularisation process by improving endothelial cell proliferation and migration.^48^ Moreover, dECM-derived from BMSCs enhances dermal fibroblast proliferation.^15^ Reseeding MSCs on naïve BMSCs-dECM or transferred BMSCs-dECM significantly enhances osteogenic differentiation ability via ERK and integrin α2β1 pathways.^49^ Meanwhile, it offers a microenvironment that retains the quality of MSCs in terms of replication, differentiation, and BMP-2 responsiveness.^32^ The BMSCs-dECM promotes calvarial bone regeneration in severe combined immunodeficiency mice.^12^ Surprisingly, cocultured HUVECs and BMSCs-dECM boost osteogenesis and angiogenesis.^50^

#### Synovium-derived stem cells

Cartilage tissue engineering has emerged as a potentially effective treatment option for cartilage repair. The creation of the cartilage-specific matrix was sustained by dECM derived from synovium-derived stem cells (SDSCs), which inhibited the expression of the enzymes that break down the matrix.^28^ Furthermore, hSDSCs-dECM promoted self-renewal ability, induced cell proliferation, and enhanced chondrogenic differentiation of SDSCs via the MAPK pathway.^51^ Therefore, dECM derived from SDSCs improves the proliferation and matrix synthesis of chondrocytes.

#### Umbilical cord MSCs

Decellularised ECM derived from Wharton's jelly MSCs (WJ-MSCs) reduces immunogenicity and improves proliferation and chondrogenic differentiation through the MAPK pathway of SDSCs and WJ-MSCs.^16^ Furthermore, dECM-derived from WJ-MSCs rescues cardiac C-kit positive cells from oxidative stress, offering enhanced *in vivo* transplantation survival and function.^52^ MSCs grown on umbilical cord MSCs (UC-MSCs)-dECM show lower level of reactive oxygen species and higher antioxidative enzyme activity, thus enhancing resistance oxidative stress.^53^^,^^54^ These properties of oxidative stress improvement reduce cellular senescence and promote cell proliferation.^55^ Placental MSC-dECM and hTERT-transduced cell lines share traits, supporting larger dECM production and aiding primary MSC expansion.^56^

#### Urine-derived stem cells

Human urine-derived stem cells (hUSCs) are promising due to their low cost and easy collection, making them practical cell sources for ECM production. USCs exhibit immunomodulatory properties and are versatile in differentiation.^57^ dECM derived from USCs is a relatively new area of research. In 2019, USC-dECM was studied in cartilage tissue engineering. USC-dECM supports chondrocyte proliferation and differentiation while promoting the synthesis of ECM proteins.^58^ Moreover, USCs-dECM reduces proinflammation and boosts anti-inflammation in macrophage coculture.^59^ Therefore, hUSCs-dECM has been proposed as a biomaterial for tissue regeneration. Together, this suggests that their ECM would be a better substrate for adult stem cell expansion. However, more research is required for full understanding their dECM characteristics and optimisation process for obtaining dECM.

### Effect of dECM derived from dental MSCs on cellular responses

Dental tissue is easily accessed via noninvasive procedures, allowing MSC isolation from waste tissues from routine dental treatments (eg, tooth extraction and wisdom tooth removal). Dental MSCs are the promising cell source for regenerative therapy, especially in craniofacial areas.^60^ Dental tissue-derived MSCs have a faster proliferation rate compared to other MSCs sources like bone marrow or adipose tissue, indicating that these dental tissue-derived MSCs could be a candidate MSC source for regenerative therapy.^61^^,^^62^ Sources of MSCs in the oral region have been reported, including pulp tissues of permanent teeth (dental pulp stem cells; DPSCs),^63^ remaining pulp tissues from primary teeth (stem cells from human exfoliated deciduous teeth; SHEDs),^64^ periodontal ligament (periodontal ligament stem cells; PDLSCs),^65^ gingival tissues (gingival stem cells),^66^ apical papilla (stem cells from the apical papilla; SCAPs), and dental follicle (dental follicle stem cells).^60^

**Figure 1:**
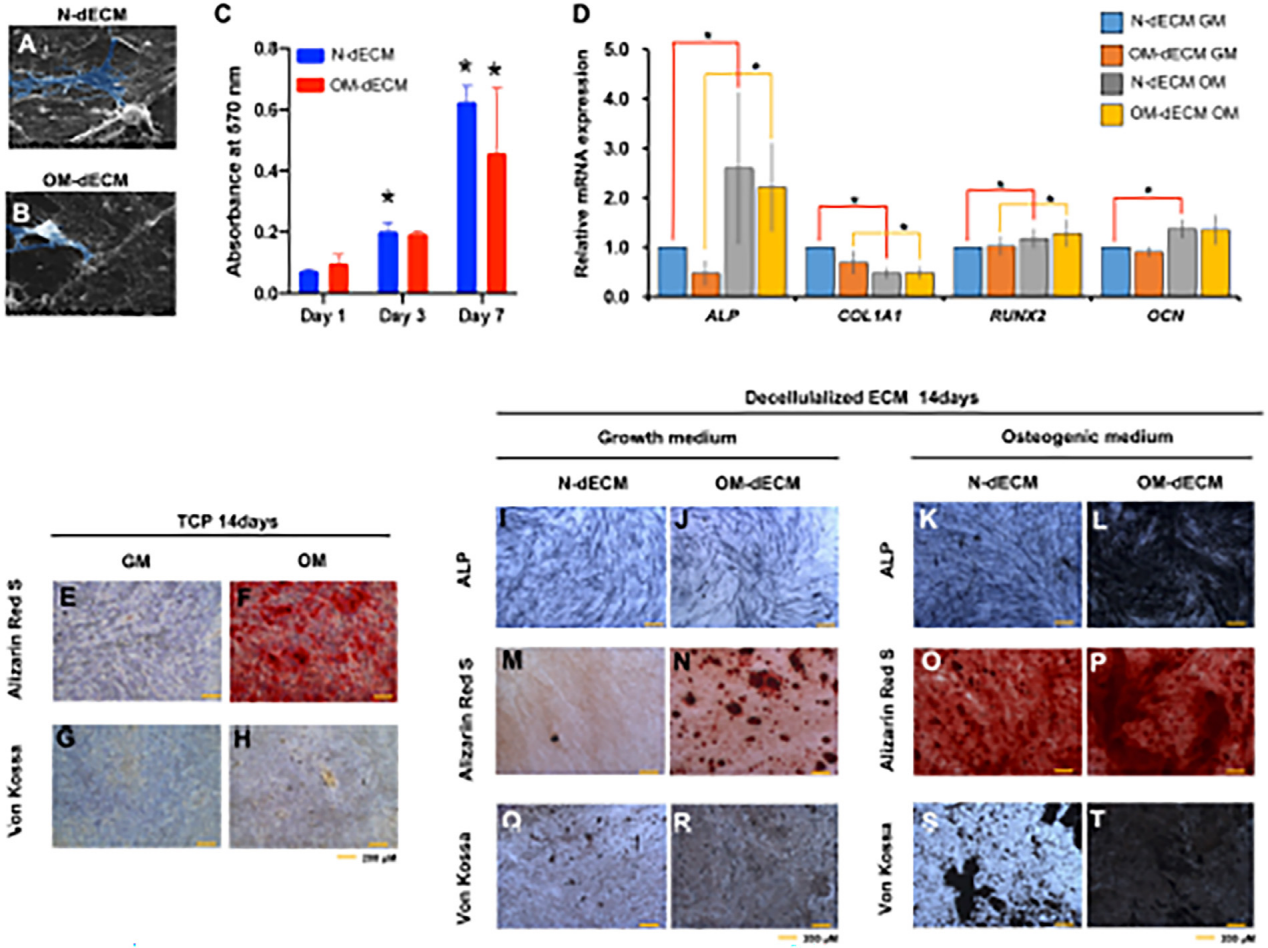
Decellularised extracellular matrix (dECM) from human dental pulp stem cells (hDPSCs) enhanced the osteogenic differentiation potency of gingival fibroblasts (GFs). GFs were reseeded on N-dECM and OM-dECM cultured with growth medium or osteogenic differentiation medium. A and B, Cell attachment was examined at 24 hours using a scanning electron microscope analysis. C, Cell metabolic activity was examined using MTT assay on days 1, 3, and 7. D, The mRNA expression of the osteogenic marker gene was evaluated using real-time quantitative PCR. GFs were seeded on TCP; after osteogenic differentiation for 14 days, ALP staining and mineral accumulation were determined using BCIP/NBT, Alizarin Red S and Von Kossa staining, respectively (E-H). GFs were reseeded on N-dECM or OM-dECM and subsequently cultured in a growth medium (I-R) or osteogenic induction medium (K-T). Cells seeded on TCP were used as the control. Asterisks indicate a statistically significant difference compared with the control (*P*-value <.05). Reprinted from Nowwarote N, Petit S, Ferre FC, et al. Extracellular matrix derived from dental pulp stem cells promotes mineralization. Front Bioeng Biotechnol 2022;9:740712, under the terms of the Creative Commons Attribution License (CC BY),^29^https://doi.org/10.3389/fbioe.2021.740712.

The ability of oral cells to adhere, proliferate, and differentiate into osteogenic differentiation can be significantly influenced by the dECM generated from dental MSCs. PDLSCs-dECM was shown to be more appropriate for osteogenic induction, while SHEDs-dECM was shown to be more appropriate for *ex vivo* growth of DPSCs.^67^ Furthermore, dECM derived from hPDLSCs, dental pulp cells, or gingival fibroblasts promotes the proliferation and osteogenic differentiation of hPDLSCs.^68^ The dECM derived from hDPSCs enriches with collagens and elastic fibres, showing the capacity for osteoinduction by improving the mineralisation of human gingival fibroblasts (Figure 1).^29^ hDPSCs-dECM demonstrated usefulness in modulating dental cell responses *in vitro*, suggesting the benefit of dental tissue engineering, mineralised tissue regeneration, or embellishing biomaterials. dECM derived from dental MSCs promotes angiogenesis, as shown by the upregulation of proangiogenic growth factors.^69^ These dECM improve the formation of dental tissues *in vivo* without the addition of exogenous factors in subcutaneous implantation.^69^ dECM derived from hDPSCs on polylactic acid (PLA) scaffolds promotes calvarial bone healing in rats, as determined by increased new bone volume and area compared to PLA alone.^70^ Interestingly, induced pluripotent stem cells (iPSCs) have been proposed as potential therapeutic cells because a patient-specific cell line can be generated. iPSCs can differentiate into numerous cell lineages. Feeder cells are required for culture during reprogramming and passage to maintain iPSCs in the stem cell stage.^71^ dECM derived from dental pulp cells is suitable as a feeder-free culture medium for dental pulp-derived iPSCs by improving iPSC attachment, cell growth, and proliferation.^72^

**Figure 2:**
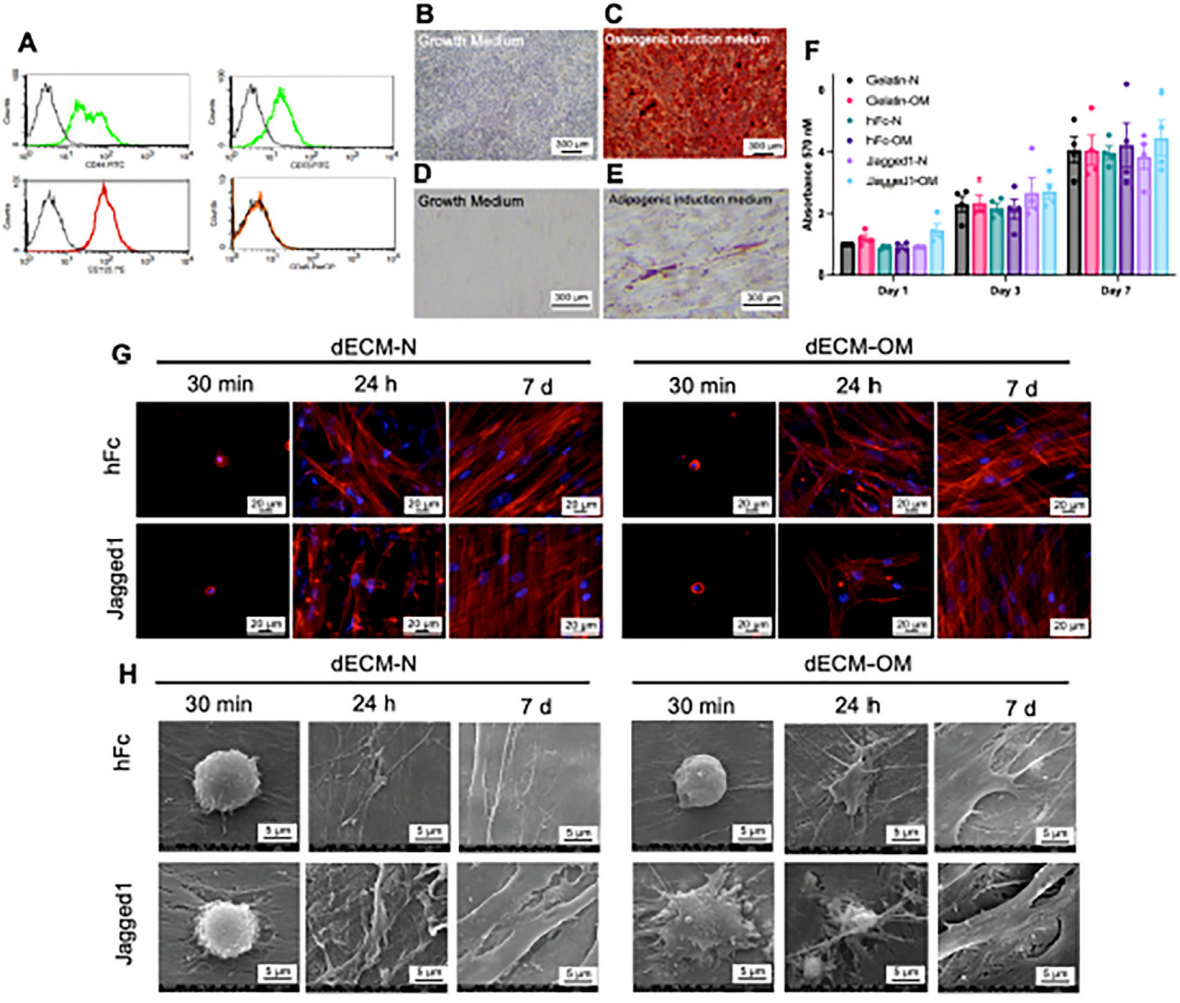
Biological responses of SCAPs on Jagged1 dECMs. Stem cells isolated from apical papilla (SCAPs) were characterised by flow cytometry to examine surface protein marker expression (A). The mineralisation was examined using Alizarin Red S staining on day 14 after osteogenic induction (B and C). The intracellular lipid accumulation was detected using Oil Red O staining on day 16 after adipogenic induction (D and E). The cell viability of SCAPs on dECM was determined using an MTT assay. The data were presented as mean ± SEM and each dot represented the value from each donor (F). Cell attachment and actin arrangement were examined using phalloidin staining at 30 minutes, 24 hours, and 7 days (G). Cell spreading was observed using scanning electron microscopic analysis (H). dECM-N, decellularised extracellular matrix derived from maintaining cells in normal medium; dECM-OM, decellularised extracellular matrix derived from maintaining cells in osteogenic medium. Reprinted from Phothichailert S, Nowwarote N, Fournier BPJ, et al. Effects of decellularized extracellular matrix derived from Jagged1-treated human dental pulp stem cells on biological responses of stem cells isolated from apical papilla. Front Cell Dev Biol 2022;10:948812, under the terms of the Creative Commons Attribution License (CC BY),^13^https://doi.org/10.3389/fcell.2022.948812.

Notch signalling activation in human dental pulp cells upregulated the ECM organisation pathway, as confirmed by RNA sequencing analysis.^73^ Proteomic and matrisome analysis of Notch ligands, Jagged-1, stimulated hDPSCs demonstrates the protein component related to osteogenic differentiation.^74^ Furthermore, the dECM of the Jagged-1 treated condition exhibited a higher mineralisation and glycosaminoglycan component compared to the dECM of the control condition. Both dECM derived from human IgG Fc fragment (hFc) and Jagged-1 treated hDPSCs support SCAPs attachment, proliferation, and osteogenic differentiation (Figure 2).^13^ However, dECM from Jagged-1-treated hDPSCs promotes higher mineralisation of reseeded SCAPs compared to dECM from the control condition. This strong evidence suggests that the manipulation of dental MSCs can alter the characteristics of the dECM and, subsequently, influence cell responses. Taking all evidence together, ECMs derived from dental cells could potentially be used as a suitable natural biomaterial scaffold for applications in the clinic, such as regenerative treatment. A summary of the effects of dECM-derived from MSCs (B) and dental MSCs (A) on cellular responses is shown in Table 3A, B.

**Table 3:** (A) Effect of the decellularised extracellular matrix derived from dental mesenchymal stem cells on cell responses.

No.	ECM derived from MSCs	ECM production medium	Decellularisation methods	Cell reseeded	Effects	Signalling pathway related	Study
1	hPDLSCs and SHEDs	10% αMEM supplement with 50 µg/mL ascorbic acid (250 uM) for 7 days	0.5% Trion X-100 containing 20 mM NH_4_OH in PBS	hDPSCs	Promote cell adhesion, cell proliferation, and osteogenic differentiation *in vitro*	-	Heng et al^67^
2	hPDLSCs and hUSCs	10% αMEM for 8 days, 80-90% confluence add 50 µg/mL ascorbic acid	0.5% Trion X-100 containing 20 mM NH_4_OH in PBS, 100 U/mL-1 DNase I	hPDLSCs	Promote cell attachment, osteogenic differentiation, adipogenic differentiation, and angiogenesis *in vitro*	-	Xiong et al^58^
3	hDPSCs	Pretreat TCP with 0.2% gelatin, culture in 10% DMEM for 21 days, add 50 µg/mL ascorbic acid on day 14 and culture in osteogenic induction medium for 21 days	0.5% Trion X-100 containing 20 mM NH_4_OH in PBS, DNase I	hGFs	Promote mineralisation *in vitro*	-	Nowwarote et al^29^
4	hDPSCs	Pretreat TCP with 0.2% gelatin, culture in 10% DMEM for 21 days, add 50 µg/mL ascorbic acid at day 14 and culture in osteogenic induction medium for 21 days	0.5% Trion X-100 containing 20 mM NH_4_OH in PBS, DNase I	hSCAPs	Promoted odonto/osteogenic differentiation *in vitro*	NOTCH	Phothichailert et al^13^

ECM, extracellular matrix; hDPSCs, human dental pulp stem cells; hGF, human gingival fibroblast; hPDLSCs, human periodontal stem cells; hUSCs, human urine derived stem cells; SCAPs, stem cells isolated from apical papilla; SHEDs, stem cell derived from human exfoliated deciduous teeth.

**Table 3:** (B) Effect of the decellularised extracellular matrix derived from mesenchymal stem cells on cell responses.

No.	ECM derived from MSCs	ECM production medium	Decellularisation methods	Cell reseeded	Effects	Signalling pathway related	Study
1	hADSCs	(1) 10%DMEM for 28 days; (2) Osteogenic medium for 28 days; (3) Adipose medium for 28 days	0.5% Trion X-100 containing 20 mM NH_4_OH in PBS, 100 U/mL DNase I, RNase I, and lipase	hADSCs	Change cell fate *in vitro*	-	Guneta et al^34^
2	hADSCs	10%DMEM for 15 days, add ascorbic acid (50 μM) at day 8	0.5% Trion X-100 containing 20 mM NH_4_OH in PBS, 100 U/mL DNase I	Retinal progenitor cells	Promote cell proliferation and neuronal differentiation *in vitro*	AKT/ERK	Ji et al^35^
3	hADSCs	Pretreat TCP with poly-L-Lysine 10% αMEM for 10 days supplement 0.2 mM ascorbic acid	1% Triton X-100 containing 20 mM NH_4_OH in PBS, 10,20, 100 U/mL	Human fibroblast and Dermal microvascular endothelial cells	Promote cell proliferation and cell adhesion *in vitro*	-	Riis et al^17^
4	hADSCs	10%DMEM for 21 days	0.5% Trion X-100 containing 25 mM NH_4_OH in PBS, 300 µg/mL DNase I	Cancer cell line (HT29 and SW480)	Successfully repopulate with cancer 3D structure and greater doxorubicin resistance *in vitro*	-	Rubi-Sans et al^36^
5	hBMSCs	Pretreat TCP with 0.2% gelatin crosslink with 1% glutaraldehyde in 1M ethanolamine, culture in 10% αMEM supplement with 100 uM ascorbic acid for 8 days	0.5% Triton X-100 containing 20 mM NH_4_OH in PBS, 100 U/mL DNase I	hBMSCs	Promote cell proliferation, osteogenic differentiation and chondrogenic differentiation. Decrease ROS and adipogenic differentiation *in vitro*	ERK1/2 and CYCLIND1	Pei et al^38^
6	hBMSCs	10,000 cells/cm^2^ in 6 well plate and serum free starvation for 3 days	0.25% Trion X-100 containing 0.25% sodium-deoxycholate in PSB, 100 µg/mL RNase, 10 U DNase	Human endothelial cells	Promote tubular morphogenesis and cell migration *in vitro*	-	Burns et al^39^
7	hBMSCs	10% αMEM supplement with 50 uM ascorbic acid for 14 days	0.5% Triton X-100 containing 20 mM NH_4_OH in PBS, 100 U/mL DNase I and digest with urea. Coat dECM on TCP	hBMSCs	Promote cell proliferation, cell attachment, cell spreading, cell migration, osteogenic differentiation, and adipogenic differentiation *in vitro*	-	Lin et al^54^
8	hBMSCs	Pretreat TCP with 0.2% gelatin crosslink with 1% glutaraldehyde in 1M ethanolamine, culture in 10% αMEM supplement with 100 uM ascorbic acid for 8 days	0.5% Triton X-100 containing 20 mM NH_4_OH in PBS, 100 U/mL DNase I	hBMSCs	Promote cell proliferation, mineralisation, and antioxidant effect of melatonin *in vitro*	-	He et al^40^
9	hBMSCs	Pretreat TCP with POAM and FB culture for 10 days in: (1) 10% DMEM; (2) 10% DMEM supplement with ascorbic acid; (3) Osteogenic differentiation medium	20 mM NH_4_OH in DDW, 1000 U/mL DNase I	Hematopoietic stem and progenitor cell (HSPCs)	Promote cell expansion *in vitro*	-	Prewitz et al^41^
10	hBMSCs	10%DMEM supplement with ascorbic acid for 100% confluence	0.5% Trion X-100 containing 20 mM NH_4_OH in PBS	mADSCs	Promote cell proliferation, colony-forming unit ability, osteogenic differentiation, adipogenic differentiation, and chondrogenic differentiation *in vitro*.	-	Xiong et al^85^
11	hBMSCs	10% MEM for 10 days, after 100% confluence add 50 µg/mL of ascorbic acid	0.5% Trion X-100 containing 20 mM NH_4_OH in PBS	Chondrocyte from the human knee articular	Promote cell proliferation, cell adhesion, cell migration, and osteogenic differentiation *in vitro*	-	Yang et al^47^
12	hBMSCs	Culture in Primer-XV MSC expansion XSFM for 4 days	detach ECM, then freeze-thaw cycling with 25 mM NH4ON in PBS	hBMSCs	Promote new bone formation in mouse *in vivo*	-	Motoike et al^12^
13	hBMSCs	10%DMEM supplement with 50 µg/mL ascorbic acid for 10 days	0.5% Triton X-100 containing 20 mM NH_4_OH in PBS, 200 U/mL DNase, RNase-free	HUVEC	Promote cell proliferation *in vitro*	Proangiogenic signalling	Sears and Ghosh^18^
14	hBMSCs	Pretreat TCP with 0.2% gelatin crosslink with 1% glutaraldehyde in 1M ethanolamine, culture in 10% DMEM for 8 days	0.5% Trion X-100 containing 20 mM NH_4_OH in PBS	UCB-MSCs	Promote cell proliferation and migration *in vitro*	-	Xu et al^48^
15	hBMSCs	15% αMEM for 15 days, add ascorbic acid (50 μM) at day 8	0.5% Triton X-100 containing 20 mM NH_4_OH in PBS	hBMSCs	Promote small cell size, cell proliferation, MSC surface marker, colony formation and osteogenic differentiation *in vitro*	-	Rakian et al^32^
16	hBMSCs	Pretreat TCP with POAM and FB culture in 10% DMEM for 10 days	20 mM NH_4_OH in DDW	Hematopoietic stem and progenitor cell (HSPCs)	Promote cell expansion, cell adhesion, and cell migration *in vitro*	Integrin BETA 3	Krater et al^42^
17	hBMSCs	15% αMEM for 15 days, add ascorbic acid (100 μM) at day 8	0.5% Triton X-100 containing 20 mM NH_4_OH in PBS, 100 U/mL DNase I	hDPSCs	Promote cell adhesion and neurogenic differentiation *in vitro*	-	Laudani et al^19^
18	hBMSCs and hADSCs	15% αMEM for 15 days, add ascorbic acid (50 μM) at day 8	0.5% Triton X-100 containing 20 mM NH_4_OH in PBS	hBMSCs, hADSCs, HeLa, MCF7 and MDA-MB-231 cancer cell lines	Promote MSCs proliferation, cell spreading, osteogenic differentiation, adipogenic differentiation and no effect on cancer cells *in vitro*	-	Marinkovic et al^86^
19	hBMSCs and HUVECs	10%DMEM for 7-10 days, add ascorbic acid (50 μM) at day 8	0.5% Triton X-100 containing 20 mM NH_4_OH in PBS	hBMSCs	Promote cell proliferation, osteogenic differentiation, adipogenic differentiation, and angiogenesis *in vitro*	-	Carvalho et al^50^
20	hMSCs	10% αMEM for 15 days	0.5% Triton X-100 containing 20 mM NH_4_OH in PBS, 100 U/mL DNase I	hMSCs	Promote cell proliferation, colony-forming unit ability, osteogenic differentiation, and adipogenic differentiation *in vitro*	ERK1/2	Decaris et al^49^
21	hMSCs	10% αMEM for 15 days, add ascorbic acid (50 μM) at day 8	0.5% Triton X-100 containing 20 mM NH_4_OH in PBS, 100 U/mL DNase I	hMSCs	Promote cell proliferation, colony-forming unit ability, osteogenic differentiation, and adipogenic differentiation *in vitro*	ERK1/2 and FGF-2	Kim and Ma 2013^43^
22	hUC-MSCs	Pretreat TCP with 0.2% gelatin and crosslink with 1% glutaraldehyde in 1M ethanolamine, culture in 10% αMEM supplement with 100 uM ascorbic acid for 8 days	0.5% Trion X-100 containing 20 mM NH_4_OH in PBS, 100 U/mL DNase I	hUC-MSCs	Promote cell proliferation, antioxidative enzyme activity, and osteogenic differentiation *in vitro*	-	Liu et al^53^
23	hUC-MSCs	Pretreat TCP with 0.2% gelatin and crosslink with 1% glutaraldehyde in 1M ethanolamine, culture in 10% αMEM supplement with 100 uM ascorbic acid for 8 days	0.5% Trion X-100 containing 20 mM NH_4_OH in PBS, 100 U/mL DNase I	hUC-MSCs	Promote cell proliferation, osteogenic differentiation, and resistance to oxidative stress-induced premature senescence *in vitro*	SIRT1	Zhou et al^88^
24	hUC-MSCs	Pretreat TCP with 25 g/mL fibronectin, culture in 10% αMEM for 15 days, add ascorbic acid (50 μM) at day 8	0.5% Trion X-100 containing 20 mM NH_4_OH in PBS	Rabbit chondrocytes	Promote cell proliferation and chondrogenic differentiation *in vitro*	-	Zhang et al^30^
25	Immortal cell line from placenta derived MSCs	10% αMEM for 14 days, add ascorbic acid (50 μM) at day 3	0.5% Trion X-100 containing 20 mM NH_4_OH in PBS	hMSCs	Promote cell proliferation and osteogenic differentiation *in vitro*	-	Kusuma et al^56^
26	Immortal cell line from placenta derived MSCs	10% αMEM for 14 days, add ascorbic acid (50 μM) at day 7	0.5% Trion X-100 containing 20 mM NH_4_OH in PBS	hMSCs from placenta	Promote cell proliferation and adipogenic differentiation *in vitro*	-	Yang et al^10^
27	hNHDF, hNHAc and hMSCs	10%DMEM supplement with ascorbic acid (50 mg/mL) for 7 days	(1) 0.025% trypsin and 0.002% EDTA in PBS; (2) 0.1% Triton X-100, 10 mM Tris-HCl, pH 8.0, and 5 mM EDTA) containing 100 µg/mL DNase I and 100 µg/mL RNase A	hNHAc	Promote cell proliferation and adhesion *in vitro*	-	Hoshiba et al^84^
28	hBMSCs, hNHDF and iPS hNHDF	Pretreat TCP with Cell Matrix^TM^, culture in 10%DMEM for 100% confluence	(1) 1% Triton X-100 in dH_2_O; (2) 1% Triton X-100 in dH_2_O and followed by 150 U/mL DNase I; (3) 1% Triton X-100 containing 20 mM NH_4_OH in PBS	hNHDF	Promote cell proliferation and enhance somatic, multipotent and pluripotent lineage-specific mRNA expression *in vitro*	-	Parmaksiz et al^15^
29	Human foetal MSCs, human adult MSCs, and human neonatal dermal fibroblast	10% αMEM for 14 days, add 50 uM ascorbic acid on day 7 or 8	0.5% Trion X-100 containing 20 mM NH_4_OH in PBS, 100 U/mL-1 DNase I	hMSCs	Promote cell proliferation, osteogenic differentiation, chondrogenic differentiation, and adipogenic differentiation *ex vivo*	-	Ng et al^45^
30	Human Wharton's jelly MSCs	10% DMEM for 14 days, add 50 µg/mL ascorbic acid at day 6	0.5% Trion X-100 containing 20 mM NH_4_OH in PBS, DNase I	human cardiac c kit cells (CCs)	Inhibit oxidative stress, promote cardiogenic differentiation *in vitro*	-	Ng et al^52^
31	hSDSCs and human Wharton's jelly MSCs	Pretreat TCP with 0.2% gelatin, culture in 10% MEM:F12 for 7 days, after 90% confluence add 250 uM L-ascorbic acid phosphate	0.5% Trion X-100 containing 20 mM NH_4_OH in PBS	hSDSCs and human Wharton's jelly MSCs	Promote cell adhesion, proliferation, and chondrogenic differentiation *in vitro*	MAPK	Wang et al^16^
32	hSDSCs	Pretreat TCP with 0.2% gelatin, culture in 10% αMEM supplement with ascorbic acid (250 uM) for 8 days	0.5% Trion X-100 containing 20 mM NH_4_OH in PBS	hSDSCs	Promoted cell proliferation, chondrogenic differentiation *in vitro*	MAPK	Zhang et al^51^
33	hUSCs	10%DMEM supplement with ascorbic acid (50 uM) for 8 days	0.5% Trion X-100 containing 20 mM NH_4_OH in PBS	hBMSCs	Promoted chondrogenic differentiation *in vitro*	WNT	Pei et al^59^
34	mBMSCs	Pretreat TCP with 0.2% gelatin crosslink with 1% glutaraldehyde in 1M ethanolamine, culture in 10% MEM for 8 days, after 90% confluence, add 100 uM L-ascorbic acid	0.5% Trion X-100 containing 20 mM NH_4_OH in PBS, 100 U/mL-1 DNase I	mBMM	Promote cell proliferation and inhibit osteoclast differentiation *in vitro*	-	Li et al^46^
35	mMSCs	10% DMEM for 3 days	0.5% Trion X-100 containing 20 mM NH_4_OH in PBS	Hepatocarcinoma cells	Promote cell adhesion and detoxification activity *in vitro*	-	Park et al^87^
36	Horse adipose MSCs and horse BMSCs	10%DMEM supplement with 50 uM ascorbic and chondrogenic medium for 15 days	0.5% Trion X-100 containing 20 mM NH_4_OH in PBS, 100 U/mL-1 DNase I	Horse adipose MSCs and horse bone marrow MSCs	Promote cell proliferation, osteogenic differentiation, and chondrogenic differentiation *in vitro*	-	Perez-Castrillo et al^27^
37	Rabbit synovium MSCs	Pretreat TCP with 0.2% gelatin, culture in 10%MEM for 8 days after 90% confluence, add 100 uM L-ascorbic acid	0.5% Trion X-100 containing 20 mM NH_4_OH in PBS, 100 U/mL-1 DNase I	Rabbit synovium MSCs	Promote cell proliferation, anti-inflammatory properties of chondrocyte *in vitro*	SIRT1	Yan et al^28^

hADSCs, human adipose stem cells; hBMSCs, human bone marrow stem cells; hMSCs, human mesenchymal stem cells; hNHA, human articular chondrocytes; hNHDF, human dermal fibroblasts; hSDSCs, human synovium derived stem cells; hUC-MSCs, human umbilical cord derived mesenchymal stem cells; hUSCs, human urine derived stem cells; HUVECs, human umbilical vein endothelial cells; mBMM, mouse bone marrow monocyte; UCB-MSCs, human umbilical cord blood mesenchymal stem cell.

### Future study on decellularised dental MSCs-derived ECM for use in regenerative dentistry

To promote regeneration in dental tissues, dental stem cells need to migrate to the injured site and proliferate. Later, these cells differentiate into mature cells or secrete growth factors, modulating biological processes to enhance regeneration. Furthermore, the biodegradation and biocompatibility of the scaffold are required to provide the appropriate physical and biological interactions. Therefore, dECM is an excellent candidate, as it can be degraded *in vivo* and is not a cytotoxic substance.^29^ Cellular responses to the composition, structure, and stiffness properties of the surrounding matrix and signalling pathways to drive the morphogenetic and pathogenic processes should be further investigated.

#### ECM scaffold combination with dental MSCs

A suitable scaffold material is vital for tissue regeneration, providing sites for cell adhesion, proliferation, and differentiation. ECM scaffolds offer the advantage of promoting natural tissue architecture regeneration in regenerative dentistry. For instance, a porcine-derived ECM scaffold seeded with DPSCs successfully regenerated dentin-pulp-like tissue with morphology, mineralisation, and mechanical properties similar to native dentin-pulp tissue.^75^ Using an ECM scaffold treated with dentin matrix protein 1 and seeded with DPSCs, the results showed the promotion of dental pulp/dentin-like tissue complex formation in both *in vitro* and *in vivo*^76^.

#### Decellularisation of dental tissue

Dental tissue decellularisation is a promising approach to the development of tissue-engineered constructs for various dental applications, such as dental pulp and periodontal regeneration. A few studies showed that decellularised bone tissue from porcine mandibles, seeded with MSCs and growth factors, can regenerate bone in the jaw.^77^ Furthermore, the potential of decellularised PDL tissue for periodontal regeneration in animal models has been demonstrated. These studies showed that decellularised PDL tissue could promote the attachment and proliferation of periodontal cells, induce the formation of new blood vessels, and support the regeneration of periodontal tissues.^78^ However, more research is necessary to evaluate the safety and efficacy of the use of decellularised dental tissues in humans. Furthermore, the development of efficient decellularisation protocols, the optimisation of scaffold properties, and the integration of growth factors and other bioactive molecules could enhance the regenerative potential of decellularised dental tissue.

#### dECM derived from dental MSCs

The use of cellular dECM in regenerative dentistry has gained attention in recent years due to its potential to promote tissue regeneration in the oral cavity, especially dECM derived from dental MSCs. Dental pulp tissue typically exhibits patterns of expression and distribution of ECM proteins like those of hDPSCs. The characteristics of the donor tooth affect the ECM proteins of DPSCs.^79^ The main issue with dental tissue decellularisation is that it can leave behind residual cellular debris that can trigger an immune response or cause complications during the decellularisation process.^77^^,^^78^ Therefore, dECM from culture dental MSCs can eliminate those limitations of ECM derived from tissues, potentially leading to novel and effective therapies for repairing and regenerating damaged or lost dental tissues. dECM derived from dental MSCs has shown promise in tissue engineering and regenerative medicine. It was shown to promote the regeneration of bone, cartilage, and dental tissues.^13^^,^^29^^,^^58^^,^^67^ Additionally, it exhibits immunomodulatory properties that reduce inflammation and enhance tissue repair.^58^ Despite these positive findings, further research is required to optimise its properties and evaluate its clinical potential.

#### The effect of dECM-secreted growth factors in regenerative dentistry

Growth factors are molecules that can stimulate cell activities and modulate tissue repair and regeneration. dECM-secreted growth factors hold immense potential for promoting various tissue regeneration processes in dentistry. Growth factors released from the dentine matrix by mineral trioxide solutions include vascular endothelial growth factor, insulin-like growth factor I and II (IGF-I, IGFBP-1), macrophage colony-stimulating factor (M-CSF), granulocyte-macrophage colony-stimulating factor, and neurotrophic growth factors. These growth factors induce proliferation and chemotaxis in dental pulp cells.^80^ In a recent investigation, it was demonstrated that dECM derived from DPSCs, enriched with IGF-binding proteins 2, 4, and 5, TGF-binding proteins 1, 2, 3, and 4, and TGF-β binding proteins, encompasses growth factor-binding proteins found within glycoproteins.^29^

Hence, the direct accumulation and functional implications of growth factors secreted by dECM derived from MSCs remain to be explored further. Understanding the signalling pathways governing oral tissue and dentin homeostasis, as well as their role in regeneration, necessitates further investigation.

#### The effects of dECM on immunomodulation in regenerative dentistry

Cell-free biomaterials, having either inflammation-boosting or calming effects, effectively control the immediate response to injury after implantation. In a xenotransplantation model, dECM derived from skeleton muscle tissue promotes the M2 macrophage phenotype and has anti-inflammatory and immunomodulatory properties.^81^ In addition, the reseeding of CD34^+^ bone marrow mononuclear cells on decellularised aortic scaffolds creates an immunomodulatory microenvironment by reduced proinflammatory cytokines (IL-8, granulocyte-macrophage colony-stimulating factor, MIP-1β, GRO-α, Entoxin, and GRO) and increased anti-inflammatory cytokines (IL-2 and TGF-β).^82^ In a study in a periodontal defect model, decellularised porcine cancellous bone treated with an anti-inflammatory substance demonstrated that the immune response in periodontal tissue began with the engagement of macrophages and giant cells. The presence of these components influenced the local environment and stimulated the release of regenerative factors, which in turn enhanced tissue regeneration.^83^ Further, a 3D bioprinting of dECM from dental follicle tissue powder with hydrogel demonstrates immunomodulatory effects by reducing the release of inflammatory cytokines from M1 macrophages and alleviating local inflammation in periodontal defects.^83^ In addition, dECM derived from DPSC notable release of CXCL12, which could be attributable to the regulation of DPSC homeostasis.^29^

Nevertheless, the innovative approach based on cellular-derived dECM and its unique function in maintaining MSC properties, particularly regarding immunoregulation and matrix formation, is not well explored in the current state of study. How this approach relates to tissue regeneration and its therapeutic applications is a crucial area of unmet research need.

## Conclusions

In summary, the utilisation of MSCs-derived decellularised matrices in regenerative dentistry offers a promising avenue for promoting tissue repair and regeneration in various oral and dental applications. On-going research and advancements in this field may lead to innovative therapies that enhance oral health and improve patient outcomes.

## Conflict of interest

None disclosed.
